# Supplementary material for: Single-Cell Transcriptomics of Cultured Amniotic Fluid Cells Reveals Complex Gene Expression Alterations in Human Fetuses With Trisomy 18
Source: Front Cell Dev Biol. 2022 Mar 22;10:825345. doi: 10.3389/fcell.2022.825345 (PMC8980718; doi:10.3389/fcell.2022.825345)
Supplement: Supplementary file 1 [file DataSheet2.pdf]

Supplementary materials

Single-cell transcriptomics of cultured amniotic fluid cells reveals complex gene expression alterations in human fetuses with trisomy 18

Jing Wang[1,2], Zixi Chen[3], Fei He[4], Trevor Lee[5], Wenjie Cai[6], Wanhua Chen[7], Nan Miao[1], Zhiwei Zeng[1], Ghulam Hussain[8], Qingwei Yang[9], Qiwei Guo[10,#] and Tao Sun[1,#]

[1]Center for Precision Medicine, School of Medicine and School of Biomedical Sciences, Huaqiao University, Xiamen, Fujian 361021, China

[2]College of Materials Science & Engineering, Huaqiao University, Xiamen, Fujian 361021, China

[3]Shenzhen Key Laboratory of Marine Bioresource and Eco- environmental Science, Shenzhen Engineering Laboratory for Marine Algal Biotechnology, Guangdong Provincial Key Laboratory for Plant Epigenetics, College of Life Sciences and Oceanography, Shenzhen University, Shenzhen 518060, China

[4]Genenergy Biotechnology, Shanghai, China

[5]Department of Cell and Developmental Biology, Cornell University Weill Medical College, New York, NY 10065, USA

[6]Department of Radiation Oncology, First Hospital of Quanzhou, Fujian Medical University, Quanzhou, Fujian, China

[7]Department of Clinical Laboratory, First Hospital of Quanzhou, Fujian Medical University, Quanzhou, Fujian, China

[8]Neurochemical Biology and Genetics Laboratory, Department of Physiology, Faculty of Life Sciences, Government College University, Faisalabad, Pakistan

[9]Department of Neurology, Zhongshan Hospital, School of Medicine, Xiamen University, Xiamen, Fujian, China.

[10]United Diagnostic and Research Center for Clinical Genetics, Women and Children's Hospital, School of Medicine & School of Public Health, Xiamen University, Xiamen, Fujian, China.

[*]Corresponding authors: taosun@hqu.edu.cn, and guoqiwei@xmu.edu.cn

Supplementary Tables

Supplemental Table S1 General statistics of single-cell sequencing.

Supplemental Table S2 Gene ontology (GO) enrichment result for the original clusters.

Supplemental Table S3 Marker genes found by Seurat in the 6 sub-clusters.

Supplemental Table S4 Differentially expressed genes (DEGs) in T18-1 cells.

Supplemental Table S5 GO enrichment result for the up-regulated genes in T18-1 cells.

Supplemental Table S6 GO enrichment result for the down-regulated genes in T18-1 cells.

Supplemental Table S7 Differentially expressed genes (DEGs) in T18-2 cells.

Supplemental Table S8 Differentially expressed genes (DEGs) in T18-3 cells.

Supplemental Table S9 GO enrichment result for the up-regulated genes in T18-2 cells.

Supplemental Table S10 GO enrichment result for the down-regulated genes in T18-2 sample.

Supplemental Table S11 GO enrichment result for the up-regulated genes in T18-3 sample.

Supplemental Table S12 GO enrichment result for the down-regulated genes in T18-3 sample.

Supplemental Table S13 All up-regulated genes in three trisomy 18 cells .

Supplementary Figures

Figure S1

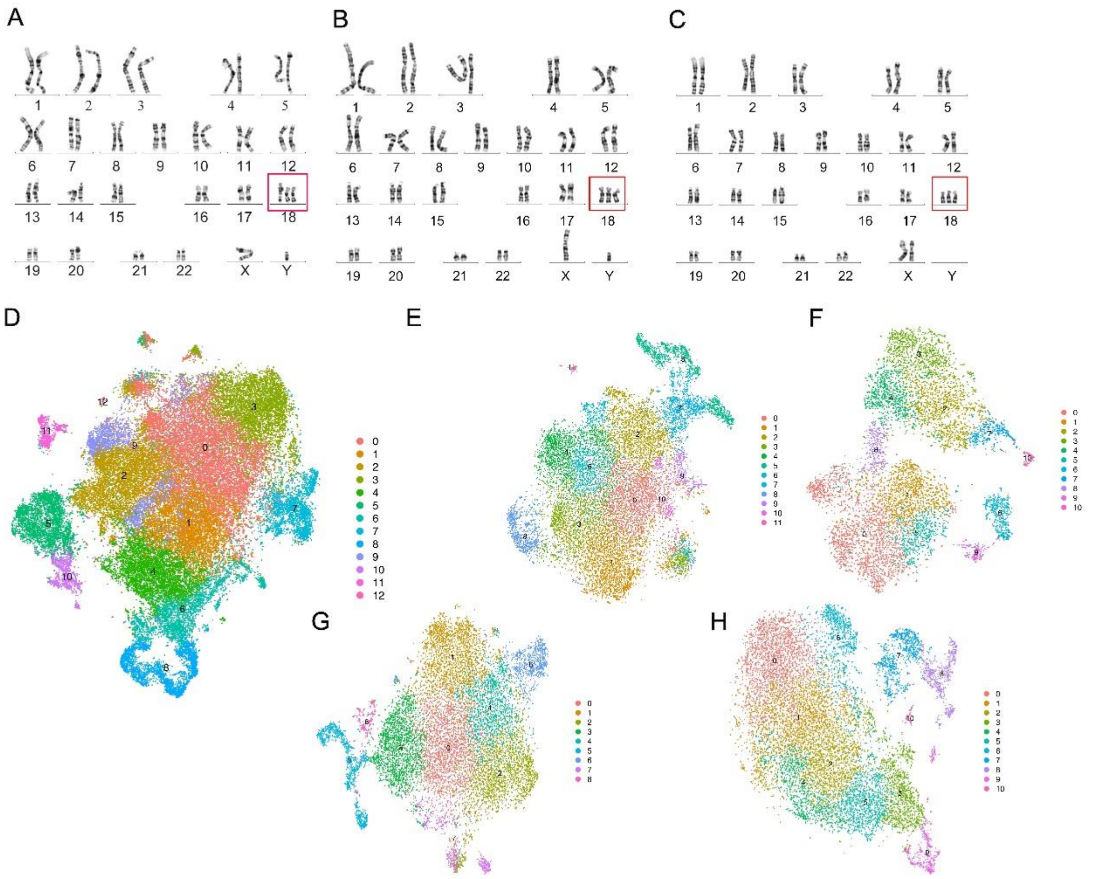

Figure S1. Functional grouping of amniotic fluid cells from euploid and trisomy 18 (T18) samples. **(A-C)** Karyotype analyses of amniotic fluid cells from trisomy 18 sample 1 (T18-1), T18-2 and T18-3. Trisomy for chromosome 18 (red box) is shown. **(D)** t-distributed Stochastic Neighbor Embedding (t-SNE) projection of all amniotic fluid cells (56,517 cells from two euploid and three trisomy 18 samples). **(E)** t-SNE projection of amniotic fluid cells from two euploid samples (18,281 cells). **(F-H)** t-SNE projections of amniotic fluid cells from T18-1 (9,092 cells) **(F)**, T18-2 (13,981 cells) **(G)**, and T18-3 (15,163 cells) **(H)** samples.

Figure S2

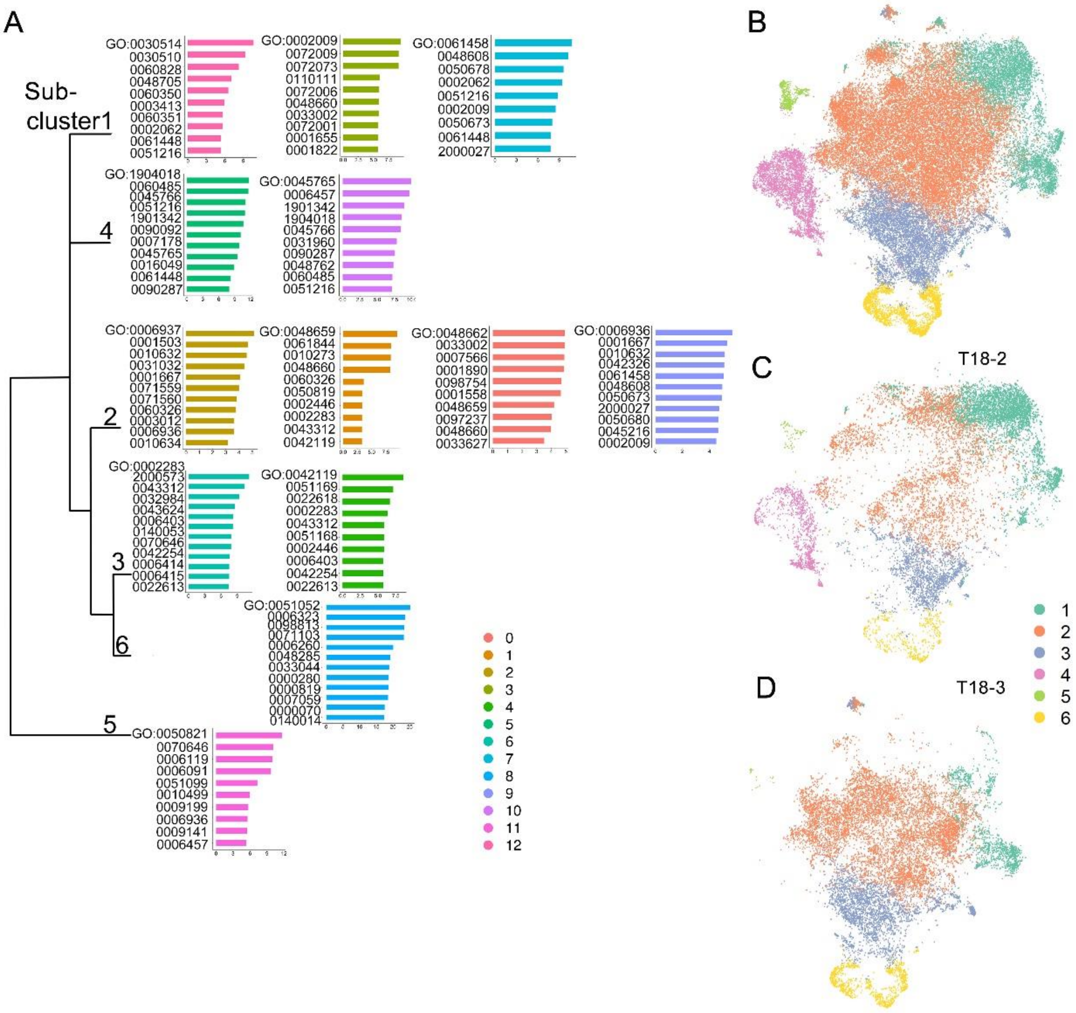

Figure S2. Functional enrichment of differentially expressed genes in amniotic fluid cells from euploid and trisomy 18 (T18) samples. **(A)** Top biological pathways enriched for differentially expressed genes identified across all analyzed cell types. Gene ontology (GO) analyses for origin-cluster with significant mRNA correlation. **(B)** t-distributed Stochastic Neighbor Embedding (t-SNE) projection of cells in euploid samples. **(C)** t-SNE projection of T18-2 sample, where each cell is grouped into one of the six clusters (distinguished by their colors). **(D)** t-SNE projection of T18-3 sample, where each cell is grouped into one of the six clusters (distinguished by their colors).

Figure S3

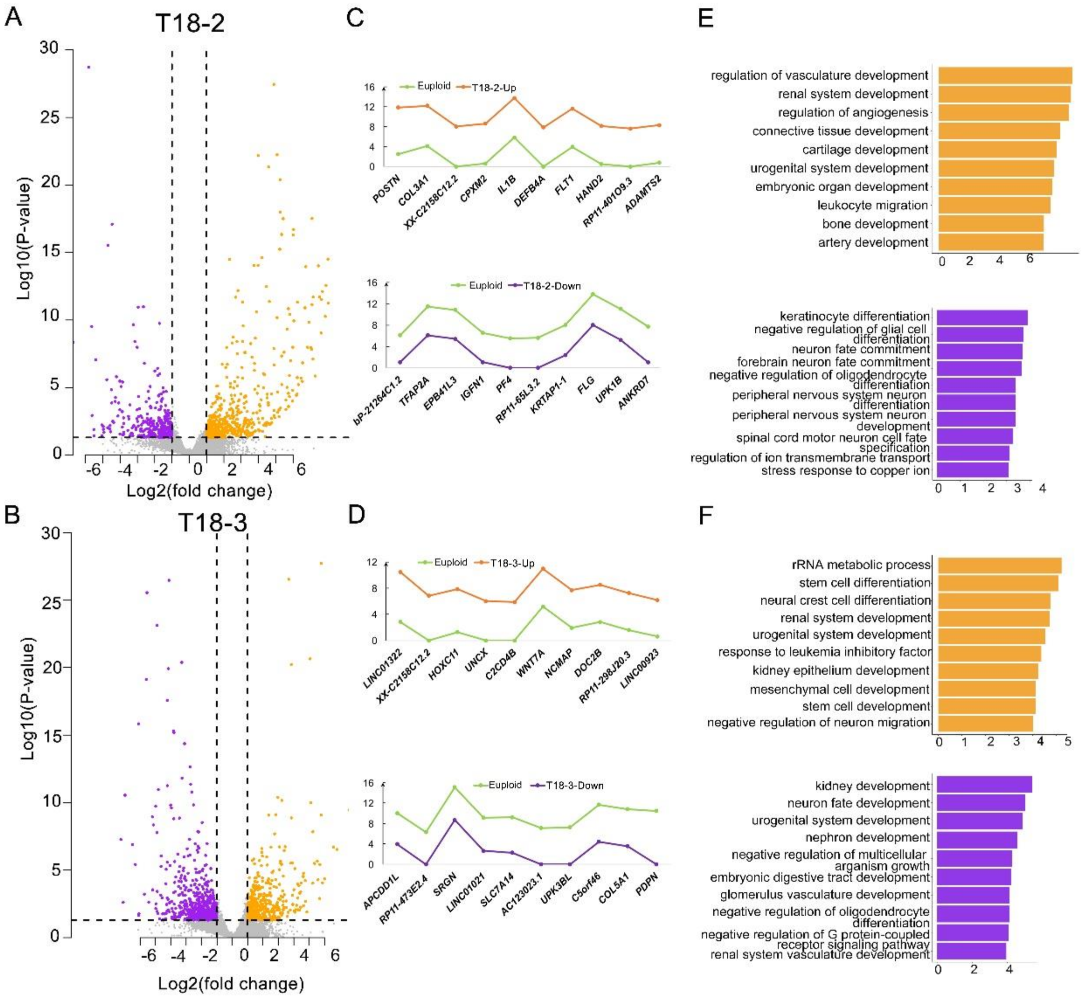

Figure S3. Altered genes in trisomy 18 amniotic fluid cells. **(A, B)** Volcano plot of differentially expressed genes by comparing the T18-2 and T18-3 samples with euploid samples. Up-regulated genes: orange dots; down-regulated genes: purple dots. **(C, D)** Top 10 (-log2fold change>1 and FDR corrected P≤0.05) up-regulated genes (orange), down-regulated genes (purple) in T18-2 and T18-3 samples compared with two euploid samples (green). **(E, F)** Gene ontology (GO) analysis of up- and down-regulated genes in T18-2 and T18-3 cells compared with two euploid samples.

Figure S4

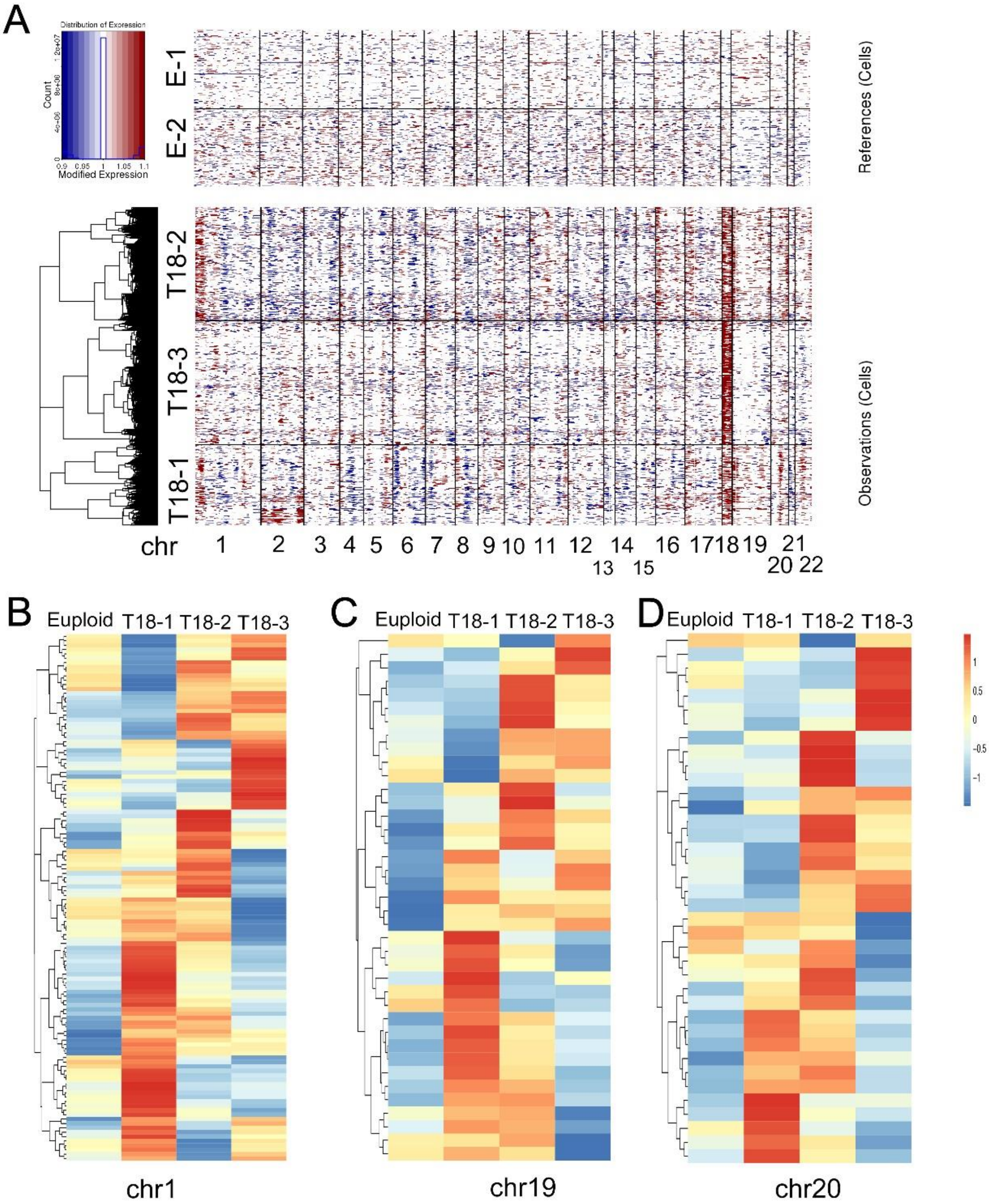

Figure S4. Trisomy 18 alters gene expression on chromosomes. **(A)** Heatmap of large-scale chromosomal copy-number variations (CNVs). Euploid cells served as reference cells, and trisomy 18 samples (T18-1, T18-2 and T 18-3) as observed cells. **(B-D)** An

increase in CNVs in genes from chromosome 1, 19 and 20 in trisomy 18 samples, compared to that in euploid samples.
